# Sexual dysfunction is highly prevalent in male survivors of malignant lymphoma

**DOI:** 10.1093/sexmed/qfad021

**Published:** 2023-05-26

**Authors:** Signe Micas Pedersen, Torsten Holm Nielsen, Anne Ortved Gang, Christian Bjørn Poulsen, Peter de Nully Brown, Niels Jørgensen, Claus Larsen Feltoft, Lars Møller Pedersen

**Affiliations:** Department of Hematology, Copenhagen University Hospital – Rigshospitalet, 2100 KBH Ø, Copenhagen, Denmark; Department of Hematology, Copenhagen University Hospital – Rigshospitalet, 2100 KBH Ø, Copenhagen, Denmark; Danish Medicines Agency, 2300 KBH S, Copenhagen, Denmark; Department of Hematology, Copenhagen University Hospital – Rigshospitalet, 2100 KBH Ø, Copenhagen, Denmark; Department of Clinical Medicine, University of Copenhagen, 2200 KBH N, Denmark; Department of Hematology, Zealand University Hospital, 4000 Roskilde, Denmark; Department of Hematology, Copenhagen University Hospital – Rigshospitalet, 2100 KBH Ø, Copenhagen, Denmark; Department of Growth and Reproduction, Copenhagen University Hospital – Rigshospitalet, 2100 KBH Ø, Denmark; Department of Endocrinology, Herlev University Hospital, 2730 Herlev, Denmark; Department of Clinical Medicine, University of Copenhagen, 2200 KBH N, Denmark; Department of Hematology, Zealand University Hospital, 4000 Roskilde, Denmark

**Keywords:** neoplasms, erectile dysfunction, sexual health, testosterone, patient reported outcome measure, quality of life, comorbidity, drug-related side effects, adverse reactions

## Abstract

**Background:**

With improved survival in patients with lymphoma, long-term toxicity and quality of life (QoL), including sexual health, have become increasingly important.

**Aim:**

We aimed to (1) determine the prevalence of erectile dysfunction (ED) in adult male lymphoma survivors; (2) determine whether testosterone deficiency, comorbidities, or lifestyle factors were associated; and (3) evaluate their impact on QoL.

**Methods:**

A cross-sectional study including 172 male survivors of Hodgkin lymphoma or diffuse large B cell lymphoma diagnosed in adulthood between 2008 and 2018 was performed. Patients were in complete metabolic remission after first-line treatment and remained in remission at follow-up (3-13 years after diagnosis). Participants completed 3 questionnaires measuring sexual health and general QoL. Serum concentrations of total testosterone were measured and thorough medical history and sociodemographic factors were obtained. The Danish SEXUS Project, European Male Ageing Study, and European Organization of Research and Treatment of Cancer (EORTC) Reference Manual were used as reference values of the general population.

**Outcomes:**

Patient reported outcome measures including the 5-item International Index of Erectile Function, EORTC C30, and EORTC 22-item Sexual Health Questionnaire.

**Results:**

ED was reported by 55.2%, which was higher than in an age-matched Danish population cohort (17.5%). Erectile function score (5-item International Index of Erectile Function) was negatively associated with comorbidity, body mass index, smoking, and age and positively with the number of children conceived before treatment and serum concentration of total testosterone. Overt testosterone deficiency in combination with ED was detected in 10 (5.7%) of 176 survivors, including excluded survivors in hormonal treatment, which is higher than for the general population (0.1%-3.2% for men <70 years of age). Mean EORTC C30 global health score for survivors with ED was lower (67.7) than for survivors without ED (80.1) but was comparable to the general population (71.2). Furthermore, a positive association was seen between sexual function and both sexual and general QoL.

**Clinical implications:**

Sexual health is important for QoL and related to comorbidities. The focus on improving QoL requires that both sexual health and comorbidities are addressed in the follow-up of lymphoma patients.

**Strengths and limitations:**

Despite the relatively high number of included survivors, the cross-sectional design of this study warrants longitudinal studies to clarify the specific underlying causes of sexual dysfunction.

**Conclusion:**

ED was highly prevalent and associated with comorbidity in lymphoma survivors, and more focus on sexual health and treatment related comorbidity is needed to improve sexual and general QoL.

## Introduction

With improved survival after treatment for malignant lymphoma,^1^ the need to manage long-term effects of disease and treatment, including sexual dysfunction (SD), is growing. Thus, SD has been described as highly prevalent affecting 20% to 54% of male survivors treated for lymphoma.^2^ However, comprehensive data on the extent, causes, and consequences of SD in long-term survivors after lymphoma treatment are scarce.

In 1992, Kornblith et al^3^ found a relationship between quality of life (QoL) and sexual health in survivors of Hodgkin lymphoma (HL). Survivors more often had decreased QoL when facing sexual health issues. SD can be a symptom of various aspects of life after cancer. Dealing with a new body image; anxiety for relapse; depression; and physical sequelae, including testosterone deficiency, neuropathy, and fatigue, can potentially affect the sexual health of cancer survivors.^4–6^

Patients might not relate sexual health issues, besides decreased fertility, to the cancer diagnosis or treatment and thereby escape diagnosis of SD. Physicians may also find the topic difficult to broach during a short outpatient visit, with other issues seeming more urgent, even though cancer patients report a need for more information about the risk of SD after treatment ^7^^,^^8^. Hence, focus on SD seems to be an unmet need of cancer survivorship. Earlier studies either have focused on SD in a cancer group of mixed diagnoses, pediatric cancer patients, both sexes or have left out important confounding factors.^8–16^ Behringer et al,^9^ compared data from 3 large randomized trials and found sexual function to be chronically impaired after late stage treatment for HL, whereas Eeltink et al^17^ found male non-HL survivors to have sexual function comparable to the general population. Because the largest study performed^9^ was designed to elucidate if there is a difference between treatment regimens and not to uncover the overall prevalence, the prevalence of SD in lymphoma survivors as an entity needs to be further elucidated. Our primary aim was to identify the extent of ED in a large population of long-term lymphoma survivors. Secondary aims were to elucidate relevant associations to testosterone level and sociodemographic and clinical factors and to evaluate whether ED affects QoL.

## Methods

### Patients

Danish-speaking male survivors of HL and diffuse large B cell lymphoma (DLBCL), treated with first-line chemotherapy between April 2008 and April 2018, with or without radiotherapy, were identified through the Danish Lymphoma Registry.^18^ First-line therapy did not change in Denmark during the treatment period. Survivors older than 18 years of age at treatment and between 18 and 65 years of age at follow-up, who were in complete metabolic remission, according to the Lugano classification, for a minimum of 1 year after completion of first line therapy, were invited to participate. Survivors treated with second-line treatment were excluded because the purpose was to investigate sexual dysfunction among a strictly homogeneous group of lymphoma patients perceived as cured of their malignant disease. Patients receiving second-line treatment have a poor prognosis and would introduce a possible confounder causing difficulties in differentiating between the independent effect of each variable on the outcome. Patients were identified from the population of the Capital Region and Region Zealand covering approximately 2.6 million citizens. Data were collected at follow-up between December 2020 and January 2022. Exclusion criteria were concurrent low-grade lymphoma, lymphoma affecting the central nervous system or testes, psychiatric disease expected to prevent compliance, prior or current abuse of anabolic steroids, current treatment of testosterone deficiency, and treatment with second-line therapy.

We performed an observational cross-sectional study. After identification, relevant survivors from the eastern part of Denmark were sent a letter of invitation. Survivors who responded were included by electronic consent. Nonresponders were called by the research team and included upon consent. Information on height, weight, exercise, smoking status, offspring status, sexual activity status, sociodemographic characteristics, Cumulative Illness Rating Scale (CIRS) scores, and confirmation of clinical data obtained from the medical record were collected by telephone interview, conducted by the same investigator for all survivors included. Sexually active meant engaging in sexual activity, either alone or with a partner, within the last 6 months. Survivors were screened for testosterone deficiency with a single measurement of serum total testosterone. Fasting blood samples were drawn before 10 am at 2 sites, Copenhagen University Hospital – Rigshospitalet and Zealand University Hospital, and analyzed at the department of clinical biochemistry at Copenhagen University Hospital – Rigshsopitalet. Analyses were performed on Cobas e801 analytical unit, with Elecsys Testosterone II assays from Roche. Reference levels used were 8.6 to 29 nmol/L for the group 18 to 49 years of age and 6.7 to 26 nmol/L for men above the age of 49 years. Values below the limit of detection was reported as <0.087 nmol/L and values above the measuring range was reported as >52.0 nmol/L. The coefficient of variation was 6%.

QoL and sexual health were measured by the validated European Organization of Research and Treatment of Cancer (EORTC) Quality of Life Questionnaire Quality of Life Questionnaire C30 version 3^19^ and EORTC 22-item Sexual Health Questionnaire (SHQ22)^20^ using the Danish versions. Sexual function was measured by 5-item International Index of Erectile Function (IIEF-5), also known as Sexual Health Inventory for Men (SHIM)^21^^,^^22^ in Danish. Description of questionnaire properties can be found in supplementary data (Table A1). Questionnaires were sent out digitally and filled in by the survivors. Comorbidity was evaluated by the CIRS score, which consists of 14 body system domains (originally 13, but adapted for elderly patients in 1991, now including hematology as a separate domain)^23^ and found to be a valid instrument.^24–26^ Each domain is scored between 0 and 4 points, resulting in a total score between 0 and 56. Zero points representing no comorbidity and 56 the highest comorbidity burden (not compatible with sustained life). Domains include cardiac; vascular; hematopoietic; respiratory; ear, nose, and throat; upper gastrointestinal tract; lower gastrointestinal tract; hepatic, renal; urethra-genital; musculoskeletal; neurologic; endocrinological; and psychiatric domains.

The Danish SEXUS Project,^27^ European Male Ageing Study,^28^ and EORTC Reference Manual^29^ were used as reference values of the general population.

Consent, questionnaires, and relevant data were registered digitally on the secure data capture platform REDCap (Research Electronic Data Capture). The study was performed in accordance with the Declaration of Helsinki and approved by the Danish Regional Committee on Health Research Ethics, Capital Region of Denmark (case number: H-20036508), and the Danish Capital Region Knowledge Center for Data Reviews (case number: P-2020-827).

**Figure 1:**
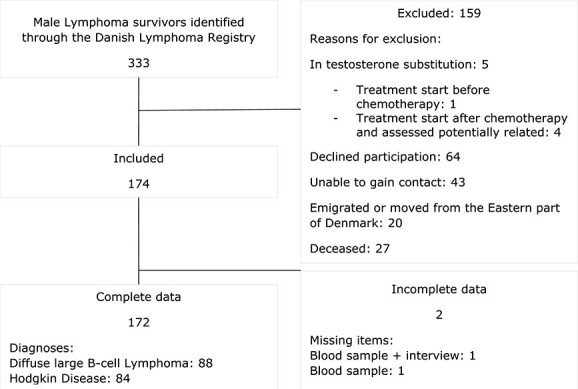
Flowchart of the study cohort.

**Figure 2:**
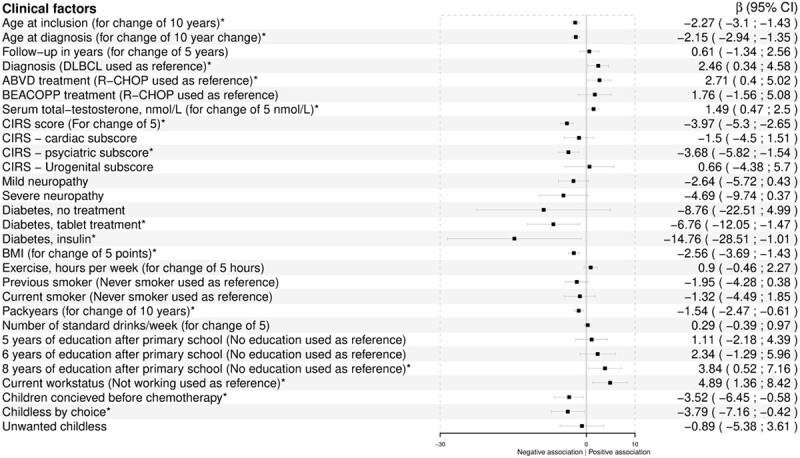
Univariate analyses by linear regression with 5-item International Index of Erectile Function (IIEF-5) score as the dependent variable. ^*^Significant association. ABVD, doxorubicin, bleomycin, vinblastine, dacarbazine; BEACOPP, Bleomycin, etoposide, doxorubicin, cyclophosphamide, oncovin, procarbazapine, prednisone; BMI, body mass index; CIRS, Cumulative Illness Rating Scale; DLBCL, diffuse large B cell lymphoma; R-CHOP, rituximab, cyclophosphamide, doxorubicin, oncovin, prednisone.

### Statistics

All statistical analyses were performed using R statistical software (version R-4.1.2; R Foundation for Statistical Computing). Descriptive statistics were used to characterize demographic data and patient treatment, using frequencies, percentages and means. *t* Tests were used to evaluate mean differences between survivors with and without ED for continuous data (dichotomized by IIEF-5 scores >21 and ≤21). Differences in categorical variables were evaluated by Fisher’s exact test. Univariate analyses were conducted using linear regression to examine the relationship between IIEF-5 scores and questionnaire scores, sociodemographics, comorbidity, and treatment variables. The analyses were done as a complete case analysis, and no missing data were present.

## Results

### Patient characteristics

Through the Danish Lymphoma Registry database, 333 survivors were identified (162 HL and 171 DLBCL), among which 238 eligible survivors were invited to participate, of which 64 declined. Thus, 174 (72.3%) survivors were initially included, and complete datasets were obtained from 172 survivors (Figure 1). There was an age difference between included and excluded survivors, with included survivors being older at diagnosis (median age 41 and 29 years of age, respectively) but younger at follow-up (median age 48 and 53 years of age, respectively). Follow-up time was not associated with erectile function in univariate analysis (Figure 2). Baseline clinical and epidemiological characteristics are outlined in Table 1 (Table A2 displays Table 1 divided by diagnoses) and treatment in Table 2.

**Table 1:** Clinical and epidemiological characteristics of 172 adult male lymphoma survivors.

Age at inclusion, y	48 (24-65)
Age at diagnosis, y	41 (19-61)
Follow-up time from diagnosis to inclusion, y	7.2 (3-13.5)
Diagnosis	
Hodgkin disease	84 (48.8)
Diffuse large B cell lymphoma	88 (51.2)
Ann Arbor stage: Hodgkin disease	
I/II	46 (54.8)
III/IV	38 (45.2)
Ann Arbor stage: diffuse large B cell lymphoma	
I/II	52 (59.1)
III/IV	36 (40.9)
Relationship status	
Married/committed relationship	144 (83.7)
Offspring status	
Child conceived before treatment	89 (51.7)
Child conceived after treatment	30 (17.4)
Childless, unwanted	14 (8.1)
Childless, by choice	39 (22.7)
Highest level of education	
0-3 y after primary school	26 (15.1)
4-5 y after primary school	58 (33.7)
6-7 y after primary school	34 (19.8)
8-9 y after primary school	54 (31.4)
Work status	
Currently employed	155 (90.1)

Values are median (range) or n (%).

**Table 2:** Treatment regimens of 84 male Hodgkin lymphoma and 88 male diffuse large B cell lymphoma survivors.

	Patients	Cycles^a^	Chemotherapy included
**Hodgkin Lymphoma**			
ABVD	58 (93.6)	4.6 (2-8)	Doxorubicin, bleomycin, vinblastine, dacarbazine
R-ABVD	1 (1.6)	6	Rituximab, doxorubicin, bleomycin, vinblastine, dacarbazine
Brentuximab-ABVD	1 (1.6)	4	Brentuximab, doxorubicin, bleomycin, vinblastine, dacarbazine
ABVD + R-CHOP	2 (3.2)	3.8 (2-5.5) + 4.0 (2-6)^*^	Doxorubicin, bleomycin, vinblastine, dacarbazine + rituximab, cyclophosphamide, doxorubicin, oncovin, prednisone
BEACOPP	10 (45.5)	6.9 (6-8)	Bleomycin, etoposide, doxorubicin, cyclophosphamide, oncovin, procarbazapine, prednisone
BEACOPP escalated	8 (36.4)	6.8 (6-8)	Bleomycin, etoposide, doxorubicin, cyclophosphamide, oncovin, procarbazapine, prednisone with escalated doses of etoposide, doxorubicin and cyclophosphamide
BEACOPP + ABVD	1 (4.6)	2 + 4	Bleomycin, etoposide, doxorubicin, cyclophosphamide, oncovin, procarbazapine, prednisone + doxorubicin, bleomycin, vinblastine, dacarbazine
BEACOPP escalated + ABVD	3 (13.6)	2.3 (2-3) + 2.3 (2-3)^*^	bleomycin, etoposide, doxorubicin, cyclophosphamide, oncovin, procarbazapine, prednisone with higher doses of etoposide, doxorubicin and cyclophosphamide + doxorubicin, bleomycin, vinblastine, dacarbazine
**Diffuse large B cell lymphoma**			
CHOP	1 (1.1)	6	Cyclophosphamide, doxorubicin, oncovin, prednisone
R-CHOP	54 (61.4)	5.1 (3-8)	Rituximab, cyclophosphamide, doxorubicin, oncovin, prednisone
R-CHOEP	27 (30.7)	6.1 (6-8)	Rituximab, cyclophosphamide, doxorubicin, oncovin, etoposide, prednisone
R-CHOEP + DA EPOCH-R	1 (1.1)	1 + 5	Rituximab, cyclophosphamide, doxorubicin, oncovin, etoposide, prednisone.
R-CHOP+R-CHOEP	1 (1.1)	3 + 3	Rituximab, cyclophosphamide, doxorubicin, oncovin, prednisone + rituximab, cyclophosphamide, doxorubicin, oncovin, etoposide, prednisone
R-COPE	2 (2.3)	4.5 (3-6)	Rituximab, cyclophosphamide, oncovin, prednisone, etoposide
R-CHOEP+High dose-Ara-C + high-dose MTX	1 (1.1)	7	Rituximab, cyclophosphamide, doxorubicin, oncovin, etoposide, prednisone, cytarabine, methotrexate
R-CODOX-M/IVAC	1 (1.1)	2 + 2	Rituximab, cyclophosphamide, doxorubicin, oncovin, methotrexate + rituximab, ifosfamide, etoposide, cytarabine
	Patients	Radiation (Gy)	
**Radiotherapy, yes**	76 (44.2)	30 (20-40)	
**Methotrexate, yes**	18 (10.5)	—	

Values are n (%) or mean (range), unless otherwise indicated.

Abbreviations: ABVD, doxorubicin, bleomycin, vinblastine, dacarbazine; BEACOPP, bleomycin, etoposide, doxorubicin, cyclophosphamide, oncovin, procarbazapine, prednisone; DA, dose adjusted; R-CHOP, rituximab, cyclophosphamide, doxorubicin, oncovin, prednisone.

^a^Results are stated as (first regimen mean [range]) + (second regimen mean [range]) when more than 1 chemotherapy regimen was given.

### Sociodemographic and clinical outcome characteristics

**Figure 3:**
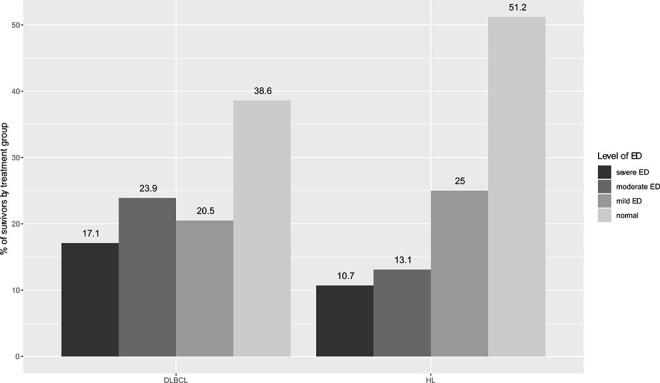
Distribution of erectile dysfunction (ED) severity in survivors with diffuse large B cell lymphoma (DLBCL) and Hodgkin lymphoma (HL) (*P* = .12).

**Figure 4:**
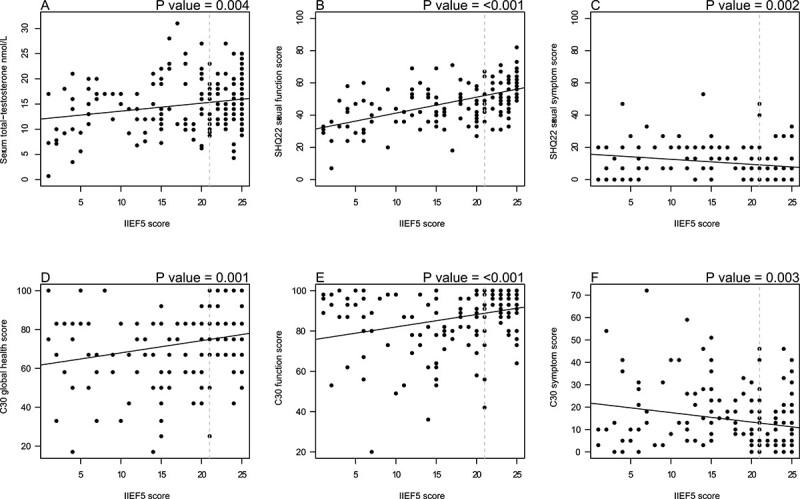
Five-item International Index of Erectile Function (IIEF-5) scores and associations with serum concentrations of total testosterone and quality-of-life (QoL) scores. (A-F) Dotted gray line representing cutoff score of 21 for erectile dysfunction. SHQ22, 22-item Sexual Health Questionnaire.

Of 172 survivors, 95 (55.2%) experienced ED: 39 (22.7%) had mild ED, 32 (18.6%) had moderate ED, and 24 (14.0%) had severe ED. The severe ED group (IIEF-5 score <8) included not only sexually active survivors with severe ED, but also sexually inactive survivors who automatically obtained a score ≤5. The influence of clinical factors on erectile function (IIEF-5 scores) is illustrated in Figure 2 and indicates that survivors of HL had a better erectile function compared with DLBCL survivors (*P =* .02). Severity of ED according to diagnosis is shown in Figure 3. A nonsignificant trend toward more severe ED could be seen among patients with DLBCL (*P =* .12). The same trend was seen when assessing sexually active survivors alone (data not shown). Survivors treated with an R-CHOP (rituximab, cyclophosphamide, doxorubicin, oncovin, prednisone)–like regimen had worse erectile function than survivors treated with ABVD (doxorubicin, bleomycin, vinblastine, dacarbazine) (*P =* .02). Eight (4.7%) survivors had serum total testosterone below the age adjusted reference level, and 6 of these reported ED (15% of men 24-39 years of age with ED, 5.9% of men 40-49 years of age with ED, 0% of men 50-59 years of age with ED, and 7.7% of men 60-65 years of age with ED) (Table 3). Despite mean serum concentration of total testosterone being within normal range for all age groups (for both survivors with and without ED), total testosterone concentration was positively associated with erectile function (*P <* .004) (Figure 4A). Survivors with ED had higher comorbidity (total CIRS) scores than survivors without ED (mean difference, 2.8; 95% CI, 1.8-3.8). While CIRS cardiac and urogenital subscores were comparable for the 2 groups, psychiatric diseases were more prevalent in the ED group (mean difference, 0.5; 95% CI, 0.2-0.8). Neuropathy and diabetes occurred equally in the 2 groups, but having diabetes severe enough to require medical treatment was associated with worse erectile function (*P <* .008). Mean body mass index (BMI) for both groups was just above the upper limit for a healthy weight and was higher for survivors with ED (mean difference, 2.7; 95% CI, 1.4-4.0; *P <* .001) (Table 3) and associated with erectile function (Figure 2). Both groups consumed the same amount of alcohol, exercised for an average of approximately 4 hours per week, and were equally often former or current smokers. However, number of pack-years was associated with erectile function (*P =* .001) (Table 3). Furthermore, good erectile function was associated with lower age (*P <* .001), long education (*P =* .02), current employment (*P <* .007), and having children before treatment (*P <* .02) (Figure 2). A small group of 5 survivors had used anabolic steroids during a maximum of 3 months 1.5 to 40 years ago. The use was sparse and hence disregarded (data not shown).

**Table 3:** Clinical outcome measures of 172 adult male lymphoma survivors.

	**Survivors with IIEF-5 score <22 (ED) (n = 95)**	**Survivors with IIEF-5 score ≥22 (No ED) (n = 77)**	**Mean difference (95% CI)**	***P* value**
Sexually active	78 (82.1)	77 (100)	—	<.001^a^
Serum total testosterone, nmol/L				
Age 24-39 y	15.1	16.1	−1.0 (−4.4 to 2.3)	—
Age 40-49 y	14.0	15.0	−1.1 (−3.9 to 2.0)	—
Age 50-59 y	15.5	15.1	0.4 (−3.3 to 4.2)	—
Age 60-65 y	12.6	16.2	−3.6 (−9.2 to 2.0)	—
Serum total testosterone below age-adjusted reference level				
Age 24-39 y	3 (15.0)	0 (0.0)	—	.05
Age 40-49 y	1 (5.9)	1 (4.3)	—	1.00
Age 50-59 y	0 (0.0)	1 (6.7)	—	.32
Age 60-65 y	2 (7.7)	0 (0.0)	—	1.00
CIRS				
Total score	9.1	6.4	2.8 (1.8 to 3.8)^a^	—
Cardiac score	2.1	1.9	0.2 (−0.5 to 0.9)	—
Psychiatric score	1.6	1.1	0.5 (0.2 to 0.8)^a^	—
Urogenital score	1.3	1.3	0.0 (−0.4 to 0.4)	—
Neuropathy				
None	72 (75.8)	68 (88.3)	—	.07
Minor, no treatment	16 (16.8)	8 (10.4)	—	
Major, requiring treatment	7 (7.4)	1 (1.3)	—	
Diabetes				
No	87 (91.6)	77 (98.7)	—	.15
Yes, no treatment	1 (1.1)	0 (0)	—	
Yes, treated with noninsulin	6 (6.3)	1 (1.3)	—	
Yes, treated with insulin	1 (1.1)	0	—	
BMI, kg/m^2^	28.8	26.1	2.7 (1.4 to 4.0)^a^	—
Hours of exercise per week	4.2	4.3	−0.1 (−1.3 to 1.1)	—
Smoking status				
Never	34 (35.8)	37 (48.1)	—	.27
Former smoker	44 (46.3)	30 (39.0)	—	
Current smoker	17 (17.9)	10 (13.0)	—	
Pack years^b^	20.2	11.9	8.3 (2.9 to 13.6)^a^	—
Alcohol use				
Intake within limits	81 (85.3)	68 (88.3)	—	.46
Above limits^c^	5 (5.3)	6 (7.8)	—	
Former abuse	9 (9.5)	3 (3.9)	—	
Alcohol, standard drinks per week^d^	5.4	6.1	−0.7 (−3.0 to 1.7)	—
	**Survivors with IIEF-5 score <22 (ED) (n = 95)**	**Survivors with IIEF-5 score ≥22 (no ED) (n = 77)**	**Mean difference (95% CI)**	**Reference scores**
EORTC SHQ22 function score	44.1	56.2	−12.1 (−15.5 to −8.8)^a^	—
EORTC SHQ22 symptom score	12.9	6.3	6.6 (4.0 to 9.2)^a^	—
EORTC C30 function score	81.8	93.8	−12.0 (−15.7 to −8.4)^a^	84.9^e^
EORTC C30 symptom score	18.0	9.1	8.9 (5.1 to 12.6)^a^	12.5^e^
EORTC C30 global health score	67.7	80.1	−12.4 (−17.5 to −7.3)^a^	71.2^e^

Values are n (%) for categorical variables with *P* values (t-tests) and mean for continuous variables with mean difference (95% CI) (Fisher’s exact test).

Abbreviations: BMI, body mass index; CIRS, Cumulative Illness Rating Scale; ED, erectile dysfunction; EORTC, European Organization of Research and Treatment of Cancer; IIEF-5, 5-item International Index of Erectile Function; SHQ22, 22-item Sexual Health Questionnaire.

^a^Significant difference.

^b^Number of packs of cigarettes smoked per day multiplied by number of years smoked.

^c^A maximum of 14 standard drinks per week.

^d^12 g of alcohol.

^e^EORTC C30 reference scores (EORTC Quality of Life Questionnaire C30 Scoring Manual).

### QoL characteristics

Sexual QoL was inferior in survivors with ED compared with sexual QoL in survivors without ED, with mean differences in sexual QoL scores (EORTC SHQ22) indicating significantly more sexual symptoms and worse sexual functioning in survivors with ED (*P <* .001) (Table 3). Furthermore, an association between erectile function and sexual QoL scores (SHQ22) was demonstrated (*P* ≤ .002) (Figure 4B, C). Mean general QoL scores (EORTC C30) for survivors with ED indicated inferior general QoL compared with both the general population^29^ and survivors without ED (*P <* .001) (Table 3), while general QoL scores for survivors without ED showed a population with good global health, a high level of functioning, and a low burden of general symptoms, with scores even better than in the general population (Table 3).^29^ Nevertheless, general QoL for all included survivors increased with higher erectile function scores (Figure 4D-4F). A comparison of sexual QoL scores (EORTC SHQ22) with general QoL scores (EORTC C30) showed that sexual health of male lymphoma survivors was more affected than general QoL (*P* ≤ .002 for comparison of both function and symptom scores) (Table 3). For survivors with ED, we found a mean C30 function score of 37.7 points higher than the mean SHQ22 function score (a difference of 37.6 points for survivors without ED). This difference was less pronounced in the symptom scores.

## Discussion

We present data from a cross-sectional study of the frequency and significance of SD in lymphoma patients presumed to be cured. We detected a high prevalence of ED in male lymphoma survivors. ED was associated with not only inferior quality of their sexual life, but also an inferior general QoL. Even though overt testosterone deficiency was only found in a small fraction of survivors, we detected a significant positive association between erectile function (IIEF-5) and testosterone level. Age, comorbidity score, smoking, and BMI was negatively associated with erectile function, whereas longer education, current employment and having had children before treatment had a positive impact on erectile function.

ED was found in 55.2% of all lymphoma survivors, and 50.3% of sexually active survivors, comparable to earlier findings.^14^^,^^15^^,^^30^^,^^31^ A comprehensive study with more than 20 000 Danish men from the general population showed that 17.5% (25-64 years of age) of sexually active men had clinical ED according to the IIEF.^27^ In our study, we investigated a wide range of possible reasons for this increased prevalence, while earlier studies had a more limited focus. Due to the cross-sectional nature of our study, we could not detect causes for ED, but they were most likely multifactorial. For instance, we detected stable social status being important for maintaining a satisfying sexual function, indicating that factors like unemployment, lack of education, and decreased fertility may contribute negatively, all of which are highly relevant after a cancer diagnosis. Sexual health is associated with a high degree of complexity and is likely dependent on social, psychological, and physical factors with complex interactions. Factors such as robustness, coping strategies, and social support networks may all play a role in influencing sexual health outcomes.

Survivors of DLBCL had poorer sexual function than HL survivors, with a trend toward more severe ED. A likely explanation is the higher age of the DLBCL cohort, which may be accompanied by a higher incidence of comorbidity.^32^ As the survivors in the present study were treated with a heterogeneous group of chemotherapy regimens, analysis of the impact of treatment was difficult due to small numbers in each cohort. However, when categorizing treatment in 3 groups, R-CHOP–like regimen, ABVD and BEACOPP (bleomycin, etoposide, doxorubicin, cyclophosphamide, oncovin, procarbazapine, prednisone), we only found treatment with an R-CHOP–like regimen to be significantly associated with inferior ED compared with ABVD. Multivariate analysis using larger cohorts of uniformly treated patients is suggested to explore the relative importance of individual factors in relation to treatment. We did not perform multivariate analyses because of small subgroups arising from our univariate analyses.

Besides social status, our data also confirmed an association between ED and overall comorbidity status. Sexual health in lymphoma patients has previously been shown to be affected by multiple factors including fatigue, mental disorders, testosterone deficiency, neuropathy, diabetes, and cardiovascular diseases.^13^^,^^15^^,^^32^^,^^33^ Furthermore, cancer survivors, including lymphoma survivors, have a higher frequency of comorbidity compared with the general population,^34^ which is probably a consequence of the lymphoma disease itself or of the treatment. Interestingly, our study only found significant association between ED and metabolic complications including high BMI and diabetes when analyzing subgroups of comorbidities. Our study did not show a significant association between cardiovascular illness, urogenital problems, neuropathy or exercise and ED, in contrast to earlier findings.^35^ This could be due to the relatively small size of the investigated subgroups. The younger age of our cohort with less comorbidity burden might also impact the lack of a significant association in our data. This is because with increasing age, comorbidity burden also increases. ED has been associated with cardiac morbidity, and even higher mortality rates compared with the general population.^36^^,^^37^ Monitoring of ED and the risk of accompanying comorbidity should therefore be included in the follow-up of lymphoma survivors.

Animal studies have shown that chemotherapeutic agents such as doxorubicin can affect the testosterone producing Leydig cells of the testes.^38^ Human studies have found a high prevalence of testosterone deficiency among survivors of lymphoma^10^^,^^39^ and testicular cancer.^40^ In our study, 8 (4.7%) of 172 survivors had serum total testosterone below the age-adjusted reference levels, with 6 (3.5%) in combination with ED. Including all relevant survivors screened for eligibility, 10 survivors suffered from testosterone deficiency and ED, corresponding to 10 (5.7%) of 176. Prevalence in the different age groups was higher than the earlier found prevalence for males from the general population (40-49 years of age: 0.1%, 50-59 years of age: 0.6% and 60-69 years of age: 3.2%)^28^ despite our cohort being younger, our lower cutoff for normal testosterone was more conservative. We also found a significant association between SD and total testosterone concentrations, indicating that even levels in the lower normal range affected sexual health, in accordance with earlier findings.^10^^,^^28^^,^^41^ Our survivors were screened with serum total testosterone alone as previously recommended.^28^ Imbalances in sex hormone binding globulin, the carrier protein of testosterone, can result in a normal serum total testosterone but a clinically relevant low serum free testosterone, although this is not highly prevalent.^28^ Thus, an altered testosterone-to-sex-hormone-binding-globulin ratio in lymphoma survivors should be considered in future studies.

Survivors in the current study had both high prevalence of ED and a decreased sexual QoL. Symptom scores were less affected than function scores, suggesting that survivors’ sexual health is influenced by other issues than symptoms alone such as psychological and social factors reported here. EORTC module for sexual health (SHQ22) has only been applied in a few studies,^42^^,^^43^ and comparison with the general population or other lymphoma groups is therefore not possible. However, it is an easy add-on module to the frequently used EORTC QLQ C30 investigating general QoL, which might increase the use in time. Interestingly, we found an association between the widely used IIEF-5 score and the SHQ22 score, suggesting that the SHQ22 is a valid tool. We also found an association between ED and general QoL. Previous research has shown a significant association between SD and health-related QoL in lymphoma survivors,^30^ and between testosterone levels and both sexual and general QoL.^28^ In our study, general QoL in lymphoma survivors without ED was not severely affected, in accordance with earlier findings.^29^ Previous studies hypothesized that cancer survivors are more appreciative of life after surviving cancer and therefore report high QoL despite of health-related problems.^44^ Survivors with ED had significantly lower QoL scores, indicating that ED was decisive for QoL in our study.

Some limitations to our study should be considered. Included survivors were older at diagnosis and had shorter mean follow-up time than excluded, which could represent selection bias. Perhaps survivors who had been in remission for many years thought their participation to be insignificant or did no longer suffer from problems. The calculated inclusion over 10 years based on incidence and inclusion criteria was slightly higher than the included cohort and may entail a risk of selection bias. However, this could also be due to underreporting in the register used to identify survivors. Inclusion and exclusion criteria were chosen to include a population representative of the general survivor in clinical practice. More stringent exclusion criteria could have resulted in a more homogeneous study population, thereby reducing the probability of confounders. However, if several factors with potential influence on sexual function had been part of the exclusion criteria, it would be contradictory to the aim of the study. Additionally, SD may be present before the cancer diagnosis and thereby not represent a newly introduced problem in cross-sectional studies like ours. Large cohort studies evaluating sexual health could identify decreasing satisfaction and function and follow the trajectory of sexual health from before the diagnosis, through the treatment and follow-up periods. However, a large longitudinal study with a required long follow-up period will be difficult to carry out. In observational studies like the current based on patient-reported outcome a risk of recall bias is present, especially for survivors with very little sexual activity. Furthermore, some questions in the patient-reported outcome measure can be difficult to answer for survivors who only engaged in sexual activity with themselves (eg, whether erections were rigid enough for penetration). This could introduce bias because results are based on the survivors’ assessment. Furthermore, ED can be a consequence of a wide range of health issues, such as pituitary illness and use of medication, which are not part of the present study. It is therefore possible that a relationship between health effects and earlier treatment has been overestimated. Our data should be validated in a longitudinal prospective study including all possible causes of ED.

## Conclusion

Our data suggest that ED is a frequent problem in male long-term survivors of malignant lymphoma with an impact on sexual health and QoL. We found an association between ED and the serum concentration of total testosterone as well as the presence of morbidities at the time of follow-up. Our data suggest that monitoring SD in the follow-up of lymphoma patients merits increased awareness as part of the standard follow-up program to increase attention to potentially treatment related comorbidities and to improve sexual and general QoL.

## Supplementary Material

Flowchart_and_tables_Sexual_dysfunction_is_highly_prevalent_qfad021
