# Supplementary material for: Sexual dysfunction is highly prevalent in male survivors of malignant lymphoma
Source: Sex Med. 2023 May 26;11(2):qfad021. doi: 10.1093/sexmed/qfad021 (PMC10225470; doi:10.1093/sexmed/qfad021)
Supplement: Flowchart_and_tables_Sexual_dysfunction_is_highly_prevalent_qfad021 [file flowchart_and_tables_sexual_dysfunction_is_highly_prevalent_qfad021.docx]

**Flowchart and tables**

**Sexual dysfunction is highly prevalent in male survivors of malignant lymphoma**

Please find below flowchart, tables 1, 2, and 3 for inclusion in the manuscript,

and two supplementary tables.

**Figure 1 – Flowchart of the study cohort**

Male Lymphoma survivors identified through the Danish Lymphoma Registry

333

Included

174

Complete data

172

Diagnoses:

Diffuse large B-cell Lymphoma: 88

Hodgkin Disease: 84

Excluded: 159

Reasons for exclusion:

In testosterone substitution: 5

- Treatment start before chemotherapy: 1
- Treatment start after chemotherapy and assessed potentially related: 4

Declined participation: 64

Unable to gain contact: 43

Emigrated or moved from the Eastern part of Denmark: 20

Deceased: 27

Incomplete data

2

Missing items:

Blood sample + interview: 1

Blood sample: 1

| **Table 1 – Clinical and epidemiological characteristics of 172 adult male lymphoma survivors** | | |
| --- | --- | --- |
| Age at inclusion, median (range) |  | 48 (24-65) |
| Age at diagnose, median (range) |  | 41 (19-61) |
| Follow-up time from diagnosis to inclusion in years, median (range) |  | 7.2 (3-13.5) |
| Diagnosis, no. (%)   - Hodgkin disease - Diffuse large B-cell lymphoma |  | 84 (48.8)  88 (51.2) |
| Ann Arbor stage: Hodgkin disease, no. (%)   - I/II - III/IV |  | 46 (54.8)  38 (45.2) |
| Ann Arbor stage: Diffuse large B-cell lymphoma, no. (%)   - I/II - III/IV |  | 52 (59.1)  36 (40.9) |
| Relationship status, no. of survivors (%)   - Married/committed relationship |  | 144 (83.7) |
| Offspring status, no. of survivors (%)   - Child conceived before treatment - Child conceived after treatment - Childless, unwanted - Childless, by choice |  | 89 (51.7)  30 (17.4)  14 (8.1)  39 (22.7) |
| Highest level of education, no. of years after primary school (%)   - 0-3 - 4-5 - 6 - 8-9 |  | 26 (15.1)  58 (33.7)  34 (19.8)  54 (31.4) |
| Work status, no. of survivors (%)   - Currently employed |  | 155 (90.1) |

| **Table 2 – Treatment regimens of 84 male Hodgkin Lymphoma and 88 male Diffuse large**  **B-cell Lymphoma survivors** | | | |
| --- | --- | --- | --- |
| **Hodgkin Lymphoma** | | | |
|  | No. of patients (%) | Mean no. of  Cycles (range)* | Chemotherapy included |
| ABVD | 58 (93.6) | 4.6 (2-8) | Doxorubicin, bleomycin, vinblastine, dacarbazine |
| R-ABVD | 1 (1.6) | 6 | Rituximab, doxorubicin, bleomycin, vinblastine, dacarbazine |
| Brentuximab-ABVD | 1 (1.6) | 4 | Brentuximab, doxorubicin, bleomycin, vinblastine, dacarbazine |
| ABVD + R-CHOP | 2 (3.2) | 3.8 (2-5.5) +  4.0 (2-6)* | Doxorubicin, bleomycin, vinblastine, dacarbazine + rituximab, cyclophosphamide, doxorubicin, oncovin, prednisone |
| BEACOPP | 10 (45.5) | 6.9 (6-8) | Bleomycin, etoposide, doxorubicin, cyclophosphamide, oncovin, procarbazapine, prednisone |
| BEACOPP escalated | 8 (36.4) | 6.8 (6-8) | Bleomycin, etoposide, doxorubicin, cyclophosphamide, oncovin, procarbazapine, prednisone with escalated doses of etoposide, doxorubicin and cyclophosphamide |
| BEACOPP + ABVD | 1 (4.6) | 2 + 4 | Bleomycin, etoposide, doxorubicin, cyclophosphamide, oncovin, procarbazapine, prednisone + doxorubicin, bleomycin, vinblastine, dacarbazine |
| BEACOPP escalated + ABVD | 3 (13.6) | 2.3 (2-3) +  2.3 (2-3)* | bleomycin, etoposide, doxorubicin, cyclophosphamide, oncovin, procarbazapine, prednisone with higher doses of etoposide, doxorubicin and cyclophosphamide + doxorubicin, bleomycin, vinblastine, dacarbazine |
| **Diffuse large B-cell Lymphoma** | | | |
|  | No. of patients (%) | No. of  cycles | Chemotherapy included |
| CHOP | 1 (1.1) | 6 | Cyclophosphamide, doxorubicin, oncovin, prednisone |
| R-CHOP | 54 (61.4) | 5.1 (3-8) | Rituximab, cyclophosphamide, doxorubicin, oncovin, prednisone |
| R-CHOEP | 27 (30.7) | 6.1 (6-8) | Rituximab, cyclophosphamide, doxorubicin, oncovin, etoposide, prednisone |
| R-CHOEP + DA-EPOCH-R | 1 (1.1) | 1 + 5 | Rituximab, cyclophosphamide, doxorubicin, oncovin, etoposide, prednisone. DA = dose adjusted |
| R-CHOP+R-CHOEP | 1 (1.1) | 3 + 3 | Rituximab, cyclophosphamide, doxorubicin, oncovin, prednisone + Rituximab, cyclophosphamide, doxorubicin, oncovin, etoposide, prednisone |
| R-COPE | 2 (2.3) | 4.5 (3-6) | Rituximab, cyclophosphamide, oncovin, prednisone, etoposide |
| R-CHOEP+High dose-Ara-C+high dose-MTX | 1 (1.1) | 7 | Rituximab, cyclophosphamide, doxorubicin, oncovin, etoposide, prednisone, cytarabine, methotrexate |
| R-CODOX-M/IVAC | 1 (1.1) | 2 + 2 | Rituximab, cyclophosphamide, doxorubicin, oncovin, methotrexate + rituximab, ifosfamide, etoposide, cytarabine |
| **Radiotherapy, yes** | No. of patients (%) | Median Gray (range) |  |
|  | 76 (44.2) | 30 (20-40) |  |
| **Methotrexate, yes** | 18 (10.5) | - |  |
| *Results are stated as: (first regimen mean (range)) + (second regimen mean (range)) when more than one chemotherapy regimen was given | | | |

| **Table 3 – Clinical outcome measures of 172 adult male lymphoma survivors.**  **Values are no. (%) for categorical variables with p values (t-tests) and mean for continuous variables with mean difference (95% CI) (Fishers exact test).** | | | | | | | |
| --- | --- | --- | --- | --- | --- | --- | --- |
|  |  | **Survivors with**  **IIEF5 score <22**  **(ED)**  **(n=95)** |  | **Survivors with**  **IIEF5 score ≥22**  **(No ED)**  **(n=77)** |  | **Mean difference (95% CI)** | **P -value** |
| Sexually active, no. (%) |  | 78 (82.1) |  | 77 (100) |  | - | <0.001† |
| Serum total-testosterone, nmol/L   - Age 24-39 - Age 40-49 - Age 50-59 - Age 60-65 |  | 15.1  14.0  15.5  12.6 |  | 16.1  15.0  15.1  16.2 |  | -1.0 (-4.4-2.3)  -1.1 (-3.9-2.0)  0.4 (-3.3-4.2)  -3.6 (-9.2-2.0) | -  -  -  - |
| Serum total-testosterone below age-adjusted reference level, no (%)   - Age 24-39 - Age 40-49 - Age 50-59 - Age 60-65 |  | 3 (15.0)  1 (5.9)  0 (0.0)  2 (7.7) |  | 0 (0.0)  1 (4.3)  1 (6.7)  0 (0.0) |  | -  - | 0.05  1.00  0.32  1.00 |
| CIRS, total score |  | 9.1 |  | 6.4 |  | 2.8 (1.8-3.8)† | - |
| CIRS, cardiac score |  | 2.1 |  | 1.9 |  | 0.2 (-0.5-0.9) | - |
| CIRS, psychiatric score |  | 1.6 |  | 1.1 |  | 0.5 (0.2-0.8)† | - |
| CIRS, urogenital score |  | 1.3 |  | 1.3 |  | 0.0 (-0.4-0.4) | - |
| Neuropathy, no. (%)   - None - Minor, no treatment - Major, requiring treatment |  | 72 (75.8)  16 (16.8)  7 (7.4) |  | 68 (88.3)  8 (10.4)  1 (1.3) |  | -  -  - | 0.07 |
| Diabetes, no. (%)   - No - Yes, no treatment - Yes, treated with non-insulin - Yes, treated with insulin |  | 87 (91.6)  1 (1.1)  6 (6.3)  1 (1.1) |  | 77 (98.7)  0 (0)  1 (1.3)  0 |  | -  -  -  - | 0.15 |
| BMI |  | 28.8 |  | 26.1 |  | 2.7 (1.4-4.0)† | - |
| Hours of exercise per week |  | 4.2 |  | 4.3 |  | -0.1 (-1.3-1.1) | - |
| Smoking status, no. (%)   - Never - Former smoker - Current smoker |  | 34 (35.8)  44 (46.3)  17 (17.9) |  | 37 (48.1)  30 (39.0)  10 (13.0) |  | -  -  - | 0.27 |
| Pack years* |  | 20.2 |  | 11.9 |  | 8.3 (2.9-13.6)† | - |
| Alcohol use, no. (%)   - Intake within limits - Above limits** - Former abuse |  | 81 (85.3)  5 (5.3)  9 (9.5) |  | 68 (88.3)  6 (7.8)  3 (3.9) |  | -  -  - | 0.46 |
| Alcohol, no. of standard drinks per week*** |  | 5.4 |  | 6.1 |  | -0.7 (-3.0-1.7) | - |
|  |  | **Survivors with**  **IIEF5 score <22**  **(ED)**  **(n=95)** |  | **Survivors with**  **IIEF5 score ≥22**  **(No ED)**  **(n=77)** |  | **Mean difference**  **(95% CI)** | **Reference scores** |
| EORTC SHQ22 function score |  | 44.1 |  | 56.2 |  | -12.1  (-15.5- -8.8)† | - |
| EORTC SHQ22 symptom score |  | 12.9 |  | 6.3 |  | 6.6  (4.0-9.2)† | - |
| EORTC C30 function score |  | 81.8 |  | 93.8 |  | -12.0  (-15.7- -8.4)† | 84.9 § |
| EORTC C30 symptom score |  | 18.0 |  | 9.1 |  | 8.9  (5.1-12.6)† | 12.5 § |
| EORTC C30 global health score |  | 67.7 |  | 80.1 |  | -12.4  (-17.5- -7.3)† | 71.2 § |
| † Significant difference  ED = erectile dysfunction. IIEF5 = International Index of Erectile Function with 5 questions. CIRS=Cumulative Illness Rating Scale. BMI=Body mass index (weight in kilograms divided by the square of the height in meters. EORTC = European Organization of Research and Treatment of Cancer. C30 = Core module with 30 questions. SHQ22 = sexual health questionnaire with 22 questions. CI = confidence interval.  * No. of packs of cigarettes smoked per day multiplied by no. of years smoked  ** A maximum of 14 standard drinks per week  *** 12 g of alcohol  § EORTC C30 reference scores (EORTC QLQ C30 Scoring Manual). | | | | | | | |

**Supplementary material:**

| **Table A1 – Questionnaire properties** | | | |
| --- | --- | --- | --- |
|  | **EORTC C30** | **EORTC SHQ22** | **IIEF5 / SHIM** |
| Use | General QoL | Sexual QoL | Erectile function |
| Questions | 30 questions in total:  28 questions on a 4 point Likert scale  2 questions on a continuous 7 point scale.  15 questions on function, 13 questions on symptoms and two on global health | 20 questions in total (male responders)  20 questions on a 4 point Likert scale  16 questions on function and 4 on symptoms. | 5 questions in total  5 questions on a 6 point Likert scale  5 questions on function |
| Range of scores | 0-100 | 0-100 | 0-25 |
| Number of scores | 3 separate scores:  Global health score  Function score  Symptom score | 2 separate scores:  Function score  Symptom score | 1 score |
| Interpretation | A high score in the function and global health domain corresponds to a high level of functioning / health, where a high score in the symptom domain corresponds to a high burden of symptoms. | A high score in the function domain corresponds to a high level of functioning, where a high score in the symptom domain corresponds to a high burden of symptoms. | A higher score represents higher sexual function, and scores below 22 indicates erectile dysfunction. Mild: 17-21. Moderate: 8-16. Severe: below 8. |
| Fayers. EORTC QLQ-C30 Scoring Manual. 2001;30:1–67  Osoba D. Interpreting the significance of changes in health-related quality-of- life scores. J Clin Oncol. 1998;16(1):139–44 | | | |

| **Table A2 – Clinical and epidemiological characteristics for 172 adult male lymphoma survivors** | | | |
| --- | --- | --- | --- |
|  | **DLBCL**  **(no. 88)** | **HL**  **(no. 84)** | **P value** |
| Age at inclusion, median (range) | 54.5 (28-65) | 39.5 (24-63) | <0.001 |
| Age at diagnose, median (range) | 48 (19-61) | 30 (19-57) | <0.001 |
| Follow-up time from diagnosis to inclusion in years, median (range) | 6.7 (3.2-12.5) | 8.0 (3.0-13.5) | 0.06 |
| Ann Arbor stage: no. (%)   - I/II - III/IV | 52 (59.1)  36 (40.9) | 46 (54.8)  38 (45.2) | 0.6 |
| Relationship status, no. of survivors (%)   - Married/committed relationship | 73 (83) | 71 (84.5) | 0.09 |
| Offspring status, no. of survivors (%)   - Child conceived before treatment - Child conceived after treatment - Childless, unwanted - Childless, by choice | 62 (70.5)  8 (9.1)  5 (5.7)  13 (14.8) | 27 (32.1)  22 (26.2)  9 (10.7)  26 (31) | <0.001 |
| Highest level of education, no. of years after primary school (%)   - 0-3 - 4-5 - 6 - 8-9 | 13 (14.8)  34 (38.6)  17 (19.3)  24 (27.3) | 13 (15.5)  24 (28.6)  17 (20.2)  30 (35.7) | 0.5 |
| Work status, no. of survivors (%)   - Currently working | 76 (86.3) | 79 (94) | 0.1 |
